# Cellular mechanisms underlying response and resistance to CDK4/6 inhibitors in the treatment of hormone receptor-positive breast cancer

**DOI:** 10.1186/s13058-022-01510-6

**Published:** 2022-03-05

**Authors:** April C. Watt, Shom Goel

**Affiliations:** 1 Peter MacCallum Cancer Centre, 305 Grattan St, Melbourne, VIC 3000 Australia; 2 Sir Peter MacCallum Department of Oncology, University of Melbourne, Parkville, VIC 3052 Australia

**Keywords:** Cyclin-dependent kinase, CDK4/6, Cell cycle, Breast cancer, Combination therapy, Drug resistance

## Abstract

Pharmacological inhibitors of cyclin-dependent kinases 4 and 6 (CDK4/6) are now an established standard of care for patients with advanced hormone receptor-positive breast cancer. The canonical mechanism underlying CDK4/6 inhibitor activity is the suppression of phosphorylation of the retinoblastoma tumor suppressor protein, which serves to prevent cancer cell proliferation. Recent data suggest that these agents induce other diverse effects within both tumor and stromal compartments, which serve to explain aspects of their clinical activity. Here, we review these phenomena and discuss how they might be leveraged in the development of novel CDK4/6 inhibitor-containing combination treatments. We also briefly review the various known mechanisms of acquired resistance in the clinical setting.

## Background

Cell cycle dysregulation leading to sustained cellular proliferation is a hallmark of cancer [1]. In cancers arising from the luminal mammary epithelium, certain cell cycle regulators—the D-type cyclins and cyclin-dependent kinases 4 and 6 (CDK4/6)—are of particular importance [2–5]. Targeted and specific inhibitors of CDK4/6 have been developed, and these agents are most effective against cells from the luminal and HER2-amplified subtypes [5, 6]. In recent years, these inhibitors have revolutionized the treatment landscape for advanced hormone receptor (HR)-positive, HER2-negative breast cancer. While the traditional mainstay of treatment for this disease has been endocrine therapy (ET), acquired resistance to ET is a near inevitability, and the addition of CDK4/6 inhibitors markedly improves patient outcomes. Preclinically, it has been shown that CDK4/6 inhibitors act synergistically with ET and can overcome ET resistance [5]. These findings formed the basis of many preclinical and clinical studies of CDK4/6 inhibitors as treatment for ER-positive breast cancer and have ultimately led to their approval for clinical use [7]. Currently, three CDK4/6 inhibitors are approved and available to treat breast cancer: palbociclib, ribociclib, and abemaciclib. Despite widespread usage of these agents in the clinic, we are only just beginning to understand their complex effects within breast cancers, as preclinical studies show that these agents induce numerous phenotypes beyond cell cycle arrest [7]. Deeper insight into the mechanisms by which CDK4/6 inhibition (CDK4/6i) modifies tumor biology will be crucial if the full clinical potential of CDK4/6 inhibitors is to be realized.

## Cyclin D-CDK4/6-retinoblastoma pathway in HR-positive breast cancer

Progression through the four phases of the cell cycle is tightly regulated by a network of cyclin proteins and their partner CDKs. CDK4/6 and their partner D-type cyclins (cyclins D1, D2, and D3) specifically regulate transition from the G1 phase to the S phase. The G1/S transition is driven by E2F transcription factors that promote expression of genes required to support DNA replication in S phase. Importantly, E2F transcriptional activity is repressed by the retinoblastoma (RB) tumor suppressor protein, which (1) directly binds to and blocks the E2F transactivation domain and (2) recruits epigenetic modifiers that install repressive chromatin marks at E2F target gene promoters [8–12].

**Fig. 1:**
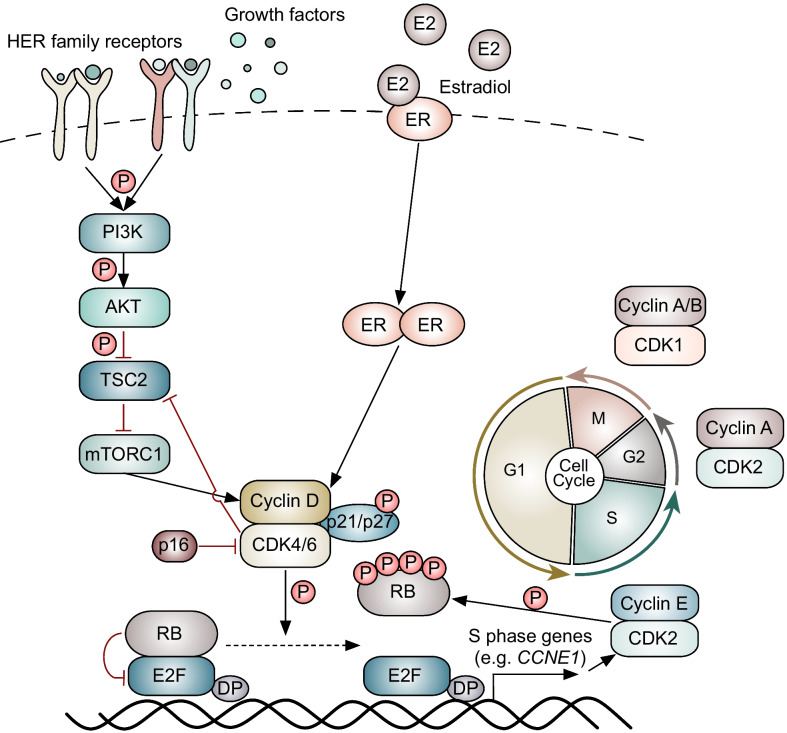
The role of cyclin D and CDK4/6 in cell cycle progression in breast cancer

The RB protein is unphosphorylated in early G1. Exposure to mitogenic growth factors at this point in the cell cycle results in a rapid rise in the level of D-type cyclins, which then bind to CDKs 4 and/or 6 (Fig. 1). The cyclin D-CDK4/6 complex then binds a third protein (either p21 or p27), and the resultant holoenzyme phosphorylates RB [13–15]. Under the classical model, CDK4/6 phosphorylates RB, inducing partial de-repression of E2F transcription factors and expression of cyclin E genes [16, 17]. Cyclin E then partners with CDK2 to hyperphosphorylate RB and establish commitment to S phase [8].

Cyclin D1 and CDK4 play particularly important roles in mammary gland biology and breast cancer. For example, cyclin D1 is required for mammary epithelial proliferation in pregnancy [18, 19], and knockout of either cyclin D1 or CDK4 prevents the development of mammary carcinomas from luminal epithelial cells driven by particular oncogenes, such as *Neu* or *Ras*, in mice [2, 3]. Cyclin D1 is also required for the maintained growth of these carcinomas [4]. Furthermore, numerous molecular features suggest that the cyclin D-CDK4/6 pathway can be hyperactivated in human HR-positive breast cancers: (1) At the genomic level, approximately 20 percent of tumors demonstrate *CCND1* gene amplification, and a smaller fraction exhibit either *CDK4* amplification (2%) or loss of *CDKN2A* (2%), which encodes for the endogenous inhibitor of CDK4/6 p16^INK4A^ [20–23]; (2) *CCND1* is also a direct transcriptional target gene of the estrogen receptor (ER), a principal driver of proliferation in HR-positive tumors [24]; and (3) activation of certain growth factor signaling pathways (most notably the PI3K-AKT-mTOR pathway) is common—whether by mutation, amplification, or increases in kinase signaling—and can either increase cyclin D levels or enhance its activity through post-translational mechanisms [25–27]. Importantly, HR-positive breast cancers also usually retain expression and function of RB, unlike triple-negative breast cancers in which RB is commonly absent or dysfunctional [5, 20, 21, 28]. Collectively, these features render CDKs 4 and 6 as attractive therapeutic targets in HR-positive breast cancer.

It is important to note that although the classical view of G1-to-S phase progression is widely accepted, the precise roles of specific CDKs in this process can be more complex. For example, certain cell types can enter S phase even in the absence of CDKs 4 and 6, including the mammary epithelial cells, and this may be due to the phosphorylation of RB by atypical cyclin D-CDK2 complexes [29, 30]. Indeed, the non-classical model of S phase entry is based upon the idea that the net phosphorylation of RB by CDK4/6 and/or CDK2 ultimately governs the G1/S transition, a concept which supports the hypothesis that CDK2 may facilitate cell cycle progression in the presence of CDK4/6 inhibitors [30, 31].

## Mechanisms of action of CDK4/6 inhibition in breast cancer: recent insights

### Cytostasis and the senescence-like state

**Fig. 2:**
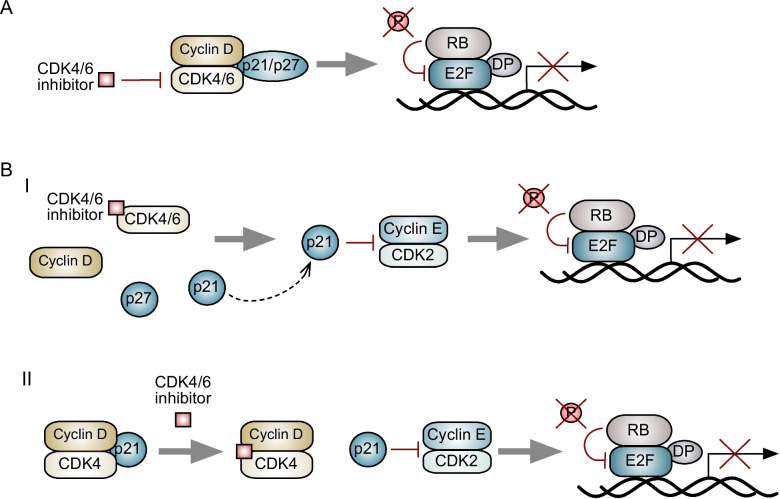
Inhibition of G1/S cyclin-dependent kinases by CDK4/6 inhibitors. **A** The traditional model suggests that CDK4/6 inhibitors inhibit active CDK4/6-cyclin D-p21/p27 holoenzymes, preventing RB phosphorylation by CDK4/6. **B** Two models suggest mechanisms by which CDK4/6 inhibitors indirectly inhibit CDK2 activity. (I) Guiley et al. propose that CDK4/6 inhibitors bind to monomeric CDK4/6, preventing the formation of CDK4/6-cyclin D-p21/p27 trimers. The free p21 then binds to and inhibits cyclin E/CDK2, preventing RB phosphorylation. This model suggests that the CDK4/6i induces cell cycle arrest through an indirect inhibition of CDK2, rather than inhibition of CDK4/6 activity. (II) Pack et al. propose that CDK4/6i inhibitors do inhibit CDK4/6 catalytic activity directly, but also displace p21 from established CDK4-cyclin D-p21 trimers, again leading to indirect inhibition of cyclin E/CDK2

The CDK4/6 inhibitors that are currently approved for treating breast cancer target the ATP-binding domains of CDKs 4 and 6 and are highly selective against these kinases [32, 33]. As one might expect given their mechanism of action, CDK4/6 inhibitors induce cytostasis (G1 cell cycle arrest) in RB-proficient luminal breast cancer cells in vitro (Fig. 2A) [4, 5, 33–36]. Given that RB is a key mediator of the senescence program, it is also not surprising that pharmacologic CDK4/6 inhibition induces a phenotype resembling senescence in luminal breast cancer cells. Multiple preclinical studies have reported a CDK4/6 inhibitor-induced “senescence-like state,” characterized by cellular enlargement and flattening, and increased β-galactosidase activity [4, 32, 33, 37, 38]. This senescent-like state is largely RB-dependent [39, 40] but might also be linked to reduced phosphorylation of the FOXM1 transcription factor and DNA methyltransferase 1 (DNMT1), both direct CDK4/6 substrates [41, 42]. It is still unclear whether CDK4/6i triggers a senescence-associated secretory phenotype (SASP) in breast cancer, or what the makeup of the SASP might be, and further study is needed to elucidate this.

Other phenotypic responses have also been reported in tumor cells, and it has been speculated that the drug effect might differ depending on the inhibitor used. Hafner et al. [43] reported that while palbociclib and ribociclib treatment induced G1 cell cycle arrest, abemaciclib arrested cells in both G1 and G2 phases, particularly at higher in vitro concentrations. They and Torres-Guzman et al. also observed that higher concentrations (greater than 0.3 µM) of abemaciclib induced apoptosis in RB-proficient breast cancer cells [35, 43]. Some of these differences might be explained by differences in the secondary targets of abemaciclib, the best validated of which are PIM kinases [38]. Some reports also describe CDK9, CDK2, and GSK3B as abemaciclib-specific targets, but unique targets of these kinases have not been conclusively shown to be hypo-phosphorylated when treating breast cancer cells at physiologically relevant concentrations [33, 38, 43–46]. On the other hand, abemaciclib and palbociclib showed similar activity (albeit with different potency) in a large panel of cancer cell lines [6], and all three CDK4/6 inhibitors exhibit greatest effect in the presence of functional RB [5, 6, 47–49]. Further study is needed to determine the extent to which the unique kinase inhibitory spectra of these agents underlie any differences in their in vitro or in vivo activity.

Finally, it is important to note that the precise pharmacologic mechanisms by which CDK4/6 inhibitors act have been the subject of recent scrutiny (Fig. 2B). Specifically, a report from Guiley et al. suggested that CDK4/6-induced cell cycle arrest is primarily a result of indirect inhibition of CDK2. Rather than binding to and inhibiting active CDK4/6-cyclin D-p21/27 trimers, CDK4/6 inhibitors were predominantly bound to inactive monomeric CDK4 or 6. As such, the inhibitors did not directly block endogenous CDK4 activity but rather prevented the formation of stable cyclin D-CDK4/6-p21/p27 complexes, leaving p21 to inhibit CDK2 activity and thus induce cell cycle arrest [50]. Pack et al. recently reported that CDK4/6 inhibitor treatment mediates two effects that work together to prevent cell cycle progression: (1) the direct inhibition of CDK4/6-mediated phosphorylation of RB, as the addition of drug leads to a decrease in RB phosphorylation within minutes, and (2) destabilization of CDK4-cyclin D-p21 trimers, allowing non-catalytic inhibition of CDK2 by p21 [51]. The latter effect was reportedly specific to CDK4 and p21 and not CDK6 and p27. Clarification of the exact mechanisms of action of CDK4/6 inhibitors will be critical, and further work is needed to elucidate the precise molecular mechanisms by which these drugs induce cell cycle arrest.

### Epigenetic remodeling

In primary fibroblasts, replicative and oncogene-induced senescence is characterized by changes in chromatin organization, including both the formation of senescence-associated heterochromatin foci (SAHF) as well as regions of enhancer activation, leading to alterations in gene transcription [52–55]. We recently reported that CDK4/6 inhibitors can induce similar changes in luminal breast cancer cells, both in vitro and in vivo (including in clinical specimens). Specifically, CDK4/6i reprograms the active enhancer landscape in an RB-dependent manner [56]. While chromatin at cell cycle gene promoters showed repressive changes upon CDK4/6i, several intergenic and intronic regions showed increased accessibility and gains in H3K27ac. These newly activated enhancers regulate processes including luminal differentiation, resistance to apoptosis, and tumor cell immunogenicity. Mechanistically, these CDK4/6i-activated enhancers are regulated by members of the activator protein-1 (AP-1) transcription factor family, as CDK4/6i increases their expression and phosphorylation [56, 57]. Consistent with this, recent studies have demonstrated that AP-1 drives chromatin accessibility and enhancer activation in benign senescent cells [58, 59]. Importantly, the extent to which ER is involved in the CDK4/6i-induced enhancer activation is yet to be determined. Further investigation is also needed to elucidate how concomitant administration of CDK4/6 inhibitors and ET modifies these epigenetic phenomena.

### Apoptotic evasion

While CDK4/6 inhibitors can induce a senescent-like state, it is not clear that these agents can directly kill luminal breast cancer cells. In fact, several preclinical and clinical studies suggest that CDK4/6i suppresses apoptosis, which is consistent with the notion that senescence is an anti-apoptotic state [60, 61]. We have recently demonstrated that in breast cancer, this “apoptosis-resistance” is underpinned in part by activation of a super-enhancer spanning the *BCL2L1* locus, which increases intracellular levels of the anti-apoptotic Bcl-2 family protein Bcl-xL. Consistent with this, Bcl-xL inhibitors restore apoptotic sensitivity in CDK4/6i-pretreated cells [56]. Similarly, Bcl-2 inhibitors can induce apoptosis in CDK4/6i-treated cells, and triplet combinations comprising ET, CDK4/6i, and venetoclax (a Bcl-2 inhibitor) are in clinical development (NCT03900884) [62]. Notwithstanding these insights, some studies report that CDK4/6i directly induces apoptosis in ER-positive breast cancer cells [35, 43]. Further work to reconcile these observations is needed to (1) better understand mechanisms of CDK4/6i-induced tumor regression and (2) design rational combinations comprising CDK4/6 inhibitors and BH3 mimetics.

### Autophagy

Autophagy and senescence are closely related, often regulated by overlapping signaling pathways. In mammary epithelial cells, kinase-active cyclin D is essential for restraining autophagy [63]. Consistent with this observation, CDK4/6i reportedly elevates various autophagic markers in ER-positive breast cancer cell lines and xenograft models. Interestingly, the addition of various inhibitors of autophagy (e.g., hydroxychloroquine) does not kill CDK4/6 inhibitor-treated breast cancer cells, but rather further enhances the senescent phenotype [64].

### Interaction with oncogenic kinase signaling circuits

Most studies searching for effective combination therapies that enhance CDK4/6i efficacy in ER-positive breast cancer have focused on concomitant inhibition of growth factor signaling pathways. Combined inhibition of CDK4/6 and growth factor receptors, such as HER2 and FGFR, or the downstream pathway members, such as PI3K, PDK1, and mTOR, has demonstrated synergy or at least heightened effects [36, 65–69]. In some cases, the effect of combined inhibition of CDK4/6 and a growth factor pathway is enhanced cytostasis or senescence [36, 65, 69], and in others, the effect is apoptosis [30, 66–68]. The combination of ET, CDK4/6, and PI3K inhibition results in maximal growth inhibition in ER-positive breast cancer models [30].

Despite the range of effective combination regimens that has been explored preclinically, the molecular mechanisms underlying their additive or synergistic effects have not been clearly delineated. One common theme is a rebound increase in activity of upstream pathways (such as the PI3K pathway) in luminal breast cancer cells treated with CDK4/6i [30, 47, 66–68]. We have also reported that CDK4/6i increases the phosphorylation of HER family receptor tyrosine kinases and AKT in luminal HER2-positive cell lines [36]. This might in part be attributed to the fact that cyclin D-CDK4/6 can phosphorylate the canonical mTOR negative regulator TSC2 [36, 70–72]. Inhibition of CDK4/6 reduces the phosphorylation of TSC2, leading to a partial reduction in mTOR activity and a rebound in upstream tyrosine kinase receptor activity [73]. While such observations might suggest heightened dependence on upstream growth factor signaling pathways in CDK4/6 inhibitor-treated cells, the downstream effects of this are not yet clear. One consequence of increased growth factor signaling is sustained stimulation of mTORC1 activity, which if uninhibited, could drive S phase progression through numerous mechanisms [74]. Another possibility is an increase in cyclin D protein levels, resulting in the formation of atypical cyclin D/CDK2 complexes that can phosphorylate RB [30].

Given these observations, combination regimens comprising inhibitors of CDK4/6 and certain growth factor pathways has moved into the clinical arena, and for two combinatorial strategies (CDK4/6-PI3K and CDK4/6-HER2), randomized phase 3 trials have already been initiated. Although initial attempts to combine CDK4/6 and PI3K inhibitors led to prohibitive toxicity [75], certain combinations have shown promise in *PIK3CA* mutant breast cancer [76], ultimately leading to the initiation of an ongoing randomized phase 3 trial exploring the benefit of adding inavolisib to the palbociclib/fulvestrant doublet (NCT04191499). In the case of HER2, numerous randomized trials are exploring the benefits of triple blockade of CDK4/6, HER2, and ER in HR-positive, HER2-positive tumors. The first of these to be reported has shown improved progression-free survival when comparing this approach to a chemotherapy-based regimen in pretreated tumors [36, 77].

### Immunogenicity

Numerous preclinical studies have reported that CDK4/6i can boost anti-tumor immune responses in models of breast and other cancers. Several distinct mechanisms underlie this phenomenon, which has been observed with all approved CDK4/6 inhibitors.

In tumor cells, CDK4/6i enhances antigen presentation on major histocompatibility complex (MHC) class I molecules in an RB-dependent manner [37, 78]. Inhibition of CDK4/6 reduces the expression of *DNMT1* (encoding DNA methyltransferase 1), an E2F target gene, resulting in hypomethylation and thus transcription of endogenous retroviral (ERV) elements [37, 79, 80]. The resultant intracellular double-stranded RNA triggers a “viral mimicry” response, characterized by interferon production and expression of interferon-stimulated genes (ISG) [37, 78]. Furthermore, we recently proposed that CDK4/6i-induced chromatin remodeling stimulates activity of enhancers overlying ERV sequences that might also drive ISG expression [56]. Recently, it has been reported that CDK4/6i can also induce metabolic stress in tumor cells, leading to expression of chemokines such as CCL5 and CXCL10 that can further enhance anti-tumor immune responses [57].

Treatment with CDK4/6 inhibitors also has a direct effect on T lymphocytes. Numerous CDK4/6 inhibitors potently suppress the proliferation of Foxp3 + regulatory T cells (T_Reg_) in the tumor microenvironment (TME), which is likely an RB-dependent phenomenon [37, 62, 81, 82]. Effector T cell function, on the other hand, can be enhanced by CDK4/6i, evidenced by enhanced effector cytokine production and reduced expression of T cell exhaustion markers [37, 78, 81]. This is attributable, at least in part, to inhibition of CDK6-mediated phosphorylation of nuclear factor of activated T cells (NFAT) transcription factors [78, 81]. Most recently, CDK4/6i has also been demonstrated to promote differentiation of CD8 T cells toward a memory cell fate, which might contribute to enhanced anti-tumor efficacy [83, 84]. Data on whether this memory differentiation effect in CD8 T cells is RB-dependent are mixed.

All told, these phenomena result in an inflamed TME and an increase in effector T cell activity, which independently contributes to the anti-tumor effects of these agents [37, 79, 81]. In an attempt to leverage this, preclinical investigators have combined several CDK4/6 inhibitors with a variety of immunotherapeutics, demonstrating superior control of tumor growth [37, 57, 78, 79, 85, 86], and the generation of T cell memory which engenders resistance to tumor re-challenge [37]. While these preclinical studies were not carried out in ER-positive breast cancer models, their results are relevant and encouraging.

Gene expression analyses of biopsies from the NeoPalAna and NeoMonarch neoadjuvant trials in luminal breast cancer suggest that this immune effect occurs in patients [37, 87], but the extent to which it can be leveraged to improve patient outcomes is still unknown. Reasons for this include that: (1) ER-positive metastatic breast cancer has thus far proven unresponsive to immune-based approaches [88, 89], and (2) early efforts to combine CDK4/6i and immuno-oncology therapy have been complicated by prohibitive toxicity [90, 91].

## Acquired resistance: mechanisms and questions

Despite the clinical success of CDK4/6i in treating ER-positive breast cancer, acquired resistance is a major clinical problem. Multiple preclinical studies have described causes for acquired resistance, and these encompass diverse mechanisms, including alterations in components of the cell cycle machinery, increased activity through oncogenic growth factor signaling pathways, metabolic changes within cancer cells, and drug-induced changes in stromal function (recently reviewed in [92]). Many of these mechanisms remain unsupported by clinical evidence, and for the sake of brevity, here we only discuss resistance mechanisms currently supported by both clinical data from breast cancer patients and preclinical evidence.

### Loss of RB function

One anticipated mechanism of resistance to CDK4/6i is loss of functional RB. The first examples were reported by Condorelli et al. [93], where acquired *RB1* mutations were detected in ER-positive breast cancer patients treated with palbociclib and fulvestrant or ribociclib and letrozole. In the PALOMA-3 study with a larger cohort of patients, whole-exome sequencing of paired circulating tumor (ct)DNA samples definitively confirmed CDK4/6 specificity of acquired *RB1* mutations, but the mutations were detected in only 5% of patients who progressed on the palbociclib and fulvestrant combination [94]. Loss of functional RB was subsequently also identified in other studies as both a feature of acquired and de novo resistance to CDK4/6i [95–97]. These findings are supported by a wealth of preclinical data showing that many CDK4/6i-mediated effects are RB-dependent.

### Other cell cycle machinery proteins

Intriguingly, several preclinical studies suggest that increased levels of CDK6 can drive resistance to CDK4/6i [98, 99]. Whether this relates to incomplete inhibition of CDK6 by the drugs [99, 100] or other kinase-independent effects of CDK6 is unclear. In ER-positive breast cancer patients, *FAT1* mutations are associated with CDK4/6i resistance, likely by increasing CDK6 expression [95].

The cyclin E/CDK2 axis has also been implicated in CDK4/6i resistance. *CCNE2* amplification has been observed in treatment-resistant tumor specimens, and overexpression of *CCNE1* mRNA is associated with poorer response to palbociclib in the metastatic setting [97, 101]. It is possible that elevations in cyclin E result in CDK2-mediated phosphorylation of RB that overcomes CDK4/6i-mediated G1 arrest [30].

### Growth factor signaling

The clinical data supporting growth factor signaling as a mechanism of CDK4/6i-resistance remains somewhat limited and is restricted to analysis of genomic alterations within tumors. It has been difficult to interpret the relevance of these data to the CDK4/6 pathway specifically, as they are almost invariably derived from patients treated with combined CDK4/6i and ET, and many of the same alterations have been implicated in ET resistance previously. One rigorous analysis comes from the PALOMA-3 trial, in which patients were randomized to receive fulvestrant with or without palbociclib. Here, ctDNA was assessed (through either whole-exome sequencing or targeted sequencing of hotspot mutation sites) in patients prior to commencing therapy and again at the time of progressive disease. In this analysis, a small number of patients’ tumors acquired mutations in *PIK3CA or FGFR2* at the time of progressive disease, but these were seen in both the control and experimental arms of the trial, making it difficult to discern the extent to which they might specifically confer CDK4/6i resistance [94].

Other data sets exploring acquired resistance are derived from cohorts of tumor biopsies studied at the time of progression on CDK4/6i, sometimes with an accompanying analysis of pre-treatment tissue. Collectively, these have shown enrichment of functional hyperactivating alterations in FGFR genes, RAS genes, *ERBB2, PTEN*, and *AKT1* in CDK4/6i resistant tumors [65, 96, 97]. However, these analyses have been limited by (1) small numbers of patients and (2) the lack of comparison to an ET-only treated cohort, and in some cases, (3) confounding by the administration of several other lines of therapy after CDK4/6i prior to acquisition of tumor tissue for analysis. Taken together, these findings suggest that genomic mutations in key growth factor receptors and signal transduction pathway members might mediate resistance to CDK4/6i in the clinic. Exactly how they drive resistance remains an open question and may be related to the ability of these pathways to drive cyclin D, RB phosphorylation, mTOR, or CDK2 [30, 65, 68, 102].

## Future directions and unanswered questions

The development of selective, potent CDK4/6 inhibitors has been a major success story for modern breast oncology, and thousands of patients have now benefited from these agents. Moving forward, key unanswered questions must be addressed with the goals of (1) enhancing the efficacy of CDK4/6 inhibitors in HR-positive breast cancer through identification of novel therapeutic combinations; (2) interrogating the plasticity of the cell cycle machinery in breast cancer as a means to understanding acquired resistance; and (3) extending the use of these agents to other breast cancer subtypes. In many cases, this will require the reassessment of assumptions that have formed the basis of much of the research in this field.

First, at a most fundamental level, research is needed to understand the molecular mechanisms of action for CDK4/6 inhibitors. It has been assumed that these agents directly inhibit CDK4/6 enzymatic activity, but this has recently been called into question by work suggesting that by binding to CDK4/6, they in fact operate as indirect CDK2 inhibitors [50]. This claim has, in turn, been refuted by other studies, and it is critical to resolve this issue, the answer to which forms the foundation for all research using these compounds [103]. Indeed, the CDK2 inhibition hypothesis is built upon the notion that CDK4/6 activity requires the formation of trimers which also contain cyclin D and p21/p27, a concept which is also controversial [103–106].

Second, more studies are needed to better understand the cellular senescence phenotype induced by CDK4/6 inhibitors. To what extent do these agents induce a SASP, and if they do, what are its components and impacts? How does the loss of p53 function, another key senescence mediator, alter the senescence phenotype? Do CDK4/6 inhibitors induce senescence in other proliferative cells within a breast tumor (e.g., fibroblasts, endothelium) and what is the impact of this? Addressing these questions adequately will require multi-omic profiling of CDK4/6 inhibitor-treated cells, sophisticated genetic modeling in vitro and in vivo, and tumor single cell profiling, and the lessons learned will likely inform novel therapeutic combinations and our thinking on drug resistance.

Third, and with specific respect to HR-positive disease, the mechanism(s) underlying synergy between CDK4/6 inhibitors and different endocrine therapies remains a poorly understood topic. In particular, the role of endocrine therapy in either enhancing or modifying the therapy-induced senescence phenotype, versus converting it to apoptosis, requires clarification. Similarly, the molecular determinants of this synergy must be studied in greater depth. Ultimately, this will shape the development of novel endocrine therapy-CDK4/6 inhibitor combinations and inform our understanding of resistance in the clinic which presumably reflects a breakdown of this synergy.

Fourth, the perennial issue of acquired CDK4/6 inhibitor resistance remains a clinical challenge, in large part because the mechanisms underlying it, and their relative frequencies, are not clear. Although preclinical studies have revealed diverse, non-genomic resistance mechanisms including altered kinase signaling, stromal cell senescence, and altered chromatin modifier function, clinical studies have almost exclusively relied on DNA sequencing of resistant cancers [92, 94, 97]. This reflects a major gap between preclinical and clinical research on this subject, which might be addressed through a more comprehensive interrogation of resistant samples including transcriptomic and epigenomic profiling at single cell resolution.

Finally, more work is needed to determine how we might exploit CDK4/6 inhibitor-mediated immunomodulation in tumors. In breast cancer, this phenomenon has gained the most traction in triple-negative disease and the results of ongoing trials in this space are eagerly awaited [107].

## Conclusion

Since CDK4/6 inhibitors were approved for use in the clinic in 2015, our understanding of their mechanisms of action has advanced significantly. We now realize that inhibiting CDK4/6 not only restrains cancer cell proliferation, but also elicits numerous diverse biological effects that can be both beneficial or harmful. This understanding should now be leveraged to inform the design of combinatorial strategies that will enhance the efficacy of CDK4/6i and tailor treatments to individual tumors. Acquired resistance remains the most pressing issue: Despite the number of studies published, a complete picture detailing common resistance mechanisms to dual CDK4/6i-ET therapy with clinical validation has yet to be constructed, and prospective, rigorously designed biospecimen collection protocols are needed to acquire pre- and post-treatment tumor samples for further study. Analysis of such samples, together with further trials, will also hopefully address the outstanding question of whether CDK4/6 inhibitors should be used after progression.

## Data Availability

Not applicable.

## Abbreviations

CDK: Cyclin-dependent kinase
RB: Retinoblastoma tumor suppressor protein
HR: Hormone receptor
ET: Endocrine therapy
CDK4/6i: CDK4/6 inhibition
PI3K: Phosphoinositide-3-kinase
AKT: Protein kinase B
mTOR: Mammalian target of rapamycin
ATP: Adenosine triphosphate
GSK3B: Glycogen synthase kinase 3 beta
ER: Estrogen receptor
FOXM1: Forkhead box protein M1
DNMT1: DNA methyltransferase 1
SASP: Senescence-associated secretory phenotype
SAHF: Senescence-associated heterochromatin foci
AP-1: Activator protein-1
FGFR: Fibroblast growth factor receptor
PDK1: Phosphoinositide-dependent kinase-1
TSC2: Tuberous sclerosis complex 2
mTORC1: Mammalian target of rapamycin complex 1
MHC: Major histocompatibility complex
ERV: Endogenous retrovirus
ISG: Interferon stimulated gene
CCL5: Chemokine ligand 5
CXCL10: C-X-C motif chemokine ligand 10
TME: Tumor microenvironment
T_reg_: Regulatory T cell
NFAT: Nuclear factor of activated T cells
ctDNA: Circulating tumor DNA
RAS: Rat sarcoma virus
